# Insights into the role of nucleotide methylation in metabolic-associated fatty liver disease

**DOI:** 10.3389/fimmu.2023.1148722

**Published:** 2023-03-20

**Authors:** Ni Zhang, Xinchen Tian, Tinghao Yan, Haochen Wang, Dengtian Zhang, Cong Lin, Qingbin Liu, Shulong Jiang

**Affiliations:** ^1^ Cheeloo College of Medicine, Shandong University, Jinan, China; ^2^ Clinical Medical Laboratory Center, Jining First People’s Hospital, Jining Medical University, Jining, China

**Keywords:** MAFLD, DNA methylation, M 6 A modification, immune microenvironment, metabolomics, epitranscriptomics

## Abstract

Metabolic-associated fatty liver disease (MAFLD) is a chronic liver disease characterized by fatty infiltration of the liver. In recent years, the MAFLD incidence rate has risen and emerged as a serious public health concern. MAFLD typically progresses from the initial hepatocyte steatosis to steatohepatitis and then gradually advances to liver fibrosis, which may ultimately lead to cirrhosis and carcinogenesis. However, the potential evolutionary mechanisms still need to be clarified. Recent studies have shown that nucleotide methylation, which was directly associated with MAFLD’s inflammatory grading, lipid synthesis, and oxidative stress, plays a crucial role in the occurrence and progression of MAFLD. In this review, we highlight the regulatory function and associated mechanisms of nucleotide methylation modification in the progress of MAFLD, with a particular emphasis on its regulatory role in the inflammation of MAFLD, including the regulation of inflammation-related immune and metabolic microenvironment. Additionally, we summarize the potential value of nucleotide methylation in the diagnosis and treatment of MAFLD, intending to provide references for the future investigation of MAFLD.

## 1 Introduction

MAFLD, formerly termed non-alcoholic fatty liver disease (NAFLD), is defined based on the coexistence of hepatic steatosis with other metabolic diseases, including obesity, type 2 diabetes and metabolic dysfunction. With the in-depth research of disease heterogeneity and pathogenesis, NAFLD was renamed MAFLD, which more accurately reflects the current understanding of the disease (1). MAFLD is the most common chronic liver disease worldwide, affecting 25% of the population (2) and up to 55.5% (3) and 90% (4) of patients with type 2 diabetes and morbid obesity, respectively. However, its specific pathogenesis still needs to be studied in depth. The “multi-hit” pathogenesis of MAFLD is now widely accepted, with insulin resistance, oxidative stress, epigenetics, lipotoxicity, inflammatory factors, altered intestinal flora, and other factors affecting hepatocyte fat content and the hepatic inflammatory milieu, thus contributing to the development of steatosis and liver inflammation (5, 6). With disease progression, MAFLD eventually advances to nonalcoholic steatohepatitis (NASH), fatty liver fibrosis, cirrhosis, and hepatocellular carcinoma (HCC) (7). There is no effective therapeutic drug for the treatment of MAFLD, although lifestyle changes, including weight loss and a balanced diet, may alleviate symptoms (8). Consequently, MAFLD has seriously impacted human health and caused a huge medical burden (9).

Epigenetics is defined as the study of the processes that modify gene expression without altering the DNA sequence directly. These processes mainly comprise nucleotide methylation, histone modification, chromosomal remodeling, and so on (10). Mounting evidence suggests that epigenetic modifications offer fresh insight into the pathophysiology of MAFLD (11–13). It has been shown that nucleotide methylation (14), histone modifications, and microRNAs (15) are involved in the pathogenesis of lipid metabolic disorder, inflammation, oxidative stress, and mitochondrial damage. Among them, DNA and RNA methylation are two of the most critical epigenetic alterations contributing to the development and progression of MAFLD (14, 16).

The immune system and inflammation also play a part in the onset of MAFLD. An inflammatory microenvironment constituted of liver resident immune cells like Kupffer cells (KCs), natural killer cells (NKCs), dendritic cells (DCs), and regulatory T lymphocytes (Tregs), characterizes NASH (17). Moreover, immune cells, working in tandem with other immune cells, can increase local inflammatory responses in the liver and exacerbate liver injury by secreting a wide variety of inflammatory factors and chemokines (18, 19). Notably, several investigations have shown that nucleotide methylation is critical in controlling the immunological response (20), suggesting that it may serve a role in the development and progression of MAFLD by triggering an inflammatory response. In addition to inflammation, the accumulation of triglycerides in liver tissue is a result of a disturbance in lipid synthesis or lipid catabolism, both of which contribute to hepatic lipid metabolic disorder (21). A growing number of studies illustrated that nucleotide methylation can impact the evolution of MAFLD through the regulation of both metabolic and immune aspects. Therefore, in this review, we aim to highlight the potential utility of regulating nucleotide methylation in diagnosing and treating MAFLD from the perspectives of the immune system and metabolism.

## 2 Background on the epigenetic function of nucleotide methylation

The accumulation of epigenetic changes, including nucleotide methylation, histone modification, non-coding RNAs, etc., is one of the fundamental processes driving cancer initiation and progression. It mainly affects the function and characteristics of genes by regulating gene transcription or translation. Nucleotide methylation, which includes DNA methylation and RNA methylation, is one of the most extensively researched epigenetic modifications.

DNA methylation, the conversion of cytosine into 5-methylcytosine (5-mC) by the addition of methyl groups provided by S-adenosyl methionine (SAM), is catalyzed by DNA methyltransferase (DNMTs) enzyme family and occurs most frequently on CpG dinucleotides. In mammals, coordinated and cooperative action between DNMTs and demethylases is essential for the regulation of DNA methylation. DNMT1 is primarily responsible for maintaining DNA methylation, while DNMT3A and DNMT3B usually carry out *de novo* methylation of unmethylated DNA or hemimethylated DNA (22). The deletion of DNMT1 and DNMT3B in mice leads to embryonic lethality, and the deletion of DNMT3A causes death within a few weeks after birth, demonstrating their vital involvement in survival and development (23). Additionally, DNMTs are intimately associated with the progression of tumors and other diseases (24). Demethylation enzymes consisting of ten-eleven translocation (TET) dioxygenases and thymine DNA glycosylase (TDG) are involved in active or passive DNA demethylation (22), and an abnormal expression of these enzymes has been linked to tumorigenesis, embryonic development and cell differentiation (25–27). In most cases, TET methylcytosine dioxygenases are responsible for the oxidation of 5-mC to 5-carboxycytosine (5caC), which is then identified and excised by TDG. Finally, the base excision repair (BER) pathway is triggered, which results in the reduction of 5-caC to unmodified cytosine. It was previously believed that DNA methylation was stable in the genome (28). However, with the development of sequencing technologies, it has been revealed that DNA methylation and demethylation are in dynamic change and balance (29, 30), which is crucial for their regulatory roles (31).

DNA methylation acts as a regulator in immune cell proliferation, differentiation, and response. Pluripotent hematopoietic stem cells can differentiate into lymphoid and myeloid stem cells. During the differentiation process, several genes of key regulatory transcription factors of myeloid stem cells, including GATA binding protein 2 (GATA2), T-cell acute leukemia 1 (TAL1), and LIM domain only 2 (LMO2), showed an increased level of DNA methylation, indicating that the differentiation of adult stem cells is regulated by DNA methylation (32). DNA methylation is intrinsically linked to cellular immunity because it is extensively reprogrammed during differentiation in functional CD8^+^ T cells. Moreover, gene expression and promoter DNA methylation are inversely correlated (33). It has been found that DNA methylation can stimulate the signaling pathways involved in the inflammatory immunological response. The expression of the zinc finger transcriptional repressor Snail can be upregulated by the Hepatitis B virus (HBV) due to an increase in mitochondrial reactive oxygen species. Together with DNMT1 and histone deacetylase 1 (HDAC1), snail binds to the promoter of suppressor of cytokine signaling 3 (SOCS3) and facilitates the epigenetic silencing of SOCS3 and prolonged activation of the IL-6/STAT3 pathway (34).

Aberrant DNA methylation is also associated with the development of cancer. Two types of DNA methylation abnormalities may exist in tumor cells: genomic hypomethylation (which enhances genomic instability and activates oncogene transcription to promote tumorigenesis), and CpG island DNA hypermethylation (which causes gene silencing and decreased expression) (35). Thus, both low and high DNA methylation levels can affect gene expression in ways that promote tumor growth. A group of genes expressed in the germline, called “cancer-testis genes” or “cancer germline genes” (CG), are usually expressed only in testicular germ cells and are silenced by DNA methylation in most somatic cells. However, aberrant activation of CG genes is a significant consequence of DNA hypomethylation in tumors. Several CG genes can trigger oncogenic pathways involved in cell proliferation and metastasis, thereby promoting tumorigenesis (36). In addition, hypermethylation of CpG islands in many genes was observed in HCC tumor samples; this process is known to repress the expression of the targeted genes. In particular, tumor growth and progression were linked to decreased expression of eyes absent 4 (EYA4) together with its hypermethylation (37).

In recent years, RNA modifications were identified to play a crucial function in regulating gene expression, with N6-methyladenosine (m^6^A) alteration accounting for approximately 80% of RNA methylation modifications. Even though the biological role of m^6^A alterations has been well reported, the effects and potential mechanisms of other RNA modifications such as N6, 2’-O-dimethyladenosine (m^6^Am), N1-methyladenosine (m^1^A), 5-methylcytosine (m^5^C), and 5-hydroxymethylcytidine (hm^5^C), remain poorly understood (16). Methylation at the N6 position of adenosine is a dynamic and reversible change that affects a wide range of biological functions (38). The regulatory proteins of m^6^A can be classified as “Writers”, “Readers” and “Erasers”, depending on their roles in the regulation processes. “Writers” are m^6^A methyltransferases mainly composed of methyltransferase-like protein 3 (METTL3) (39), methyltransferase-like protein 14 (METTL14) (40), and Wilms tumor 1 associated protein (WTAP) (41). “Readers” are m^6^A binding proteins including YT521-B homology-domain-family protein (YTHDFs) (42), insulin-like growth factor 2 mRNA binding protein (IGF2BPs), and YT521-B homology-domain-containing protein (YTHDCs) (43). “Erasers” are m^6^A demethylases, including fat mass and obesity-associated protein (FTO) (44) and alkB homolog 5 (ALKBH5) (45). They together constitute an efficient and regulatory network of m^6^A RNA modifications.

An increasing number of studies have discovered that RNA-modified m^6^A is critical in maintaining immunological homeostasis. Researchers revealed that knocking down the m^6^A methyltransferase METTL3 or METTL14 reduced the proliferative activity of CD4^+^ T cells. These findings suggest that m^6^A modification is vital for maintaining T cell homeostasis (46). METTL3-mediated m^6^A alternation has also been found to promote DC maturation by inducing cytokine production through the NF-κB signaling pathway. Furthermore, METTL3 could strengthen T cell activation by regulating the translation of CD40, CD80, and Tirap mRNA in DC cells (47).

**Figure 1:**
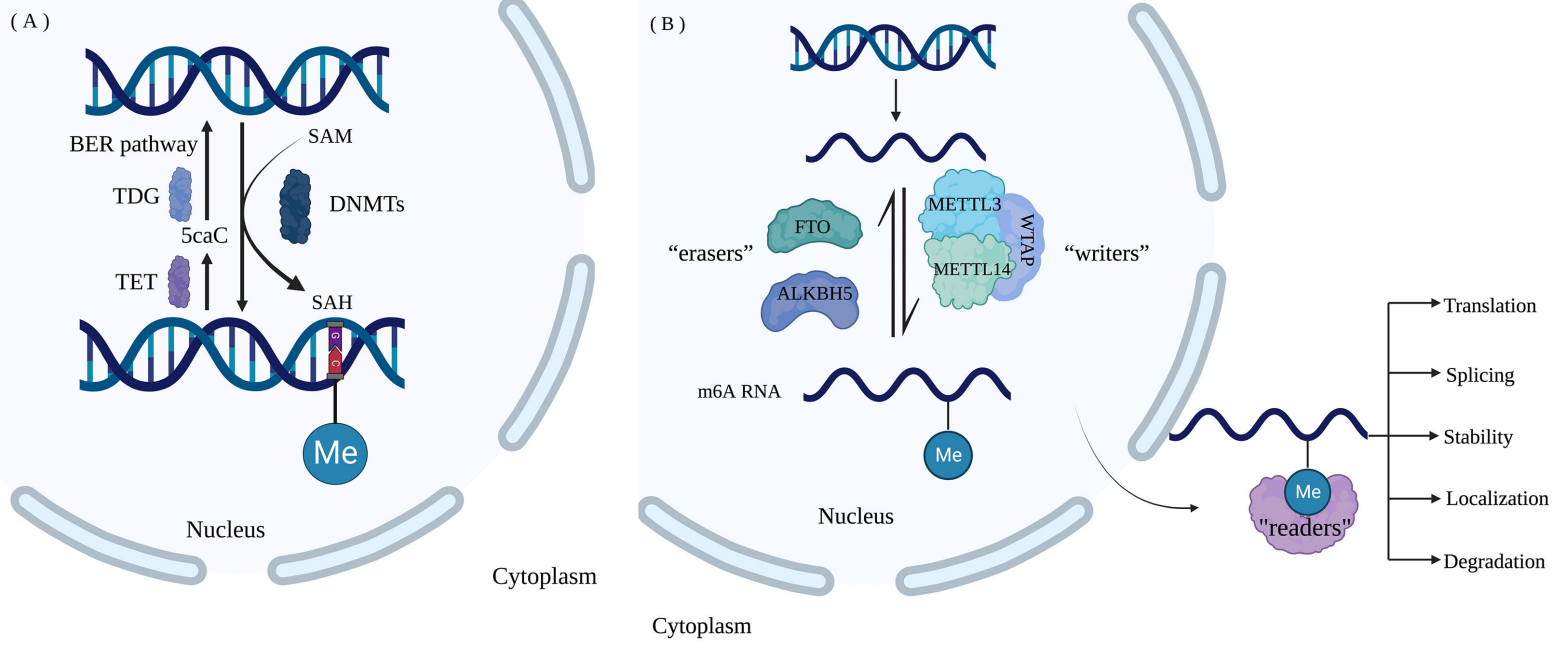
Nucleotide modification process **(A)** DNA methylation and demethylation. DNMTs catalyze the addition of methyl to C in the CG of DNA forms 5-mC, and methyl-group is provided by SAM. 5-mC undergoes oxidation by TET dioxygenase, recognition and excision by TDG and reduction to unmodified cytosine by the BER pathway. **(B)** M^6^A RNA methylation. The dynamic m^6^A modification of RNA is catalyzed by “Writers” consisting of methyltransferases METTL3, METTL14, and WTAP and is removed by “Erasers” including FTO and ALKBH5. “Readers” are m^6^A- binding proteins, including YTHDFs, IGF2BPs, and YTHDCs, regulating RNA translation, localization, splicing, degradation, and stability. (Created with BioRender.com).

There is growing evidence that m^6^A modification also affects carcinogenesis and tumor growth. m^6^A methylation can influence cancer cell metastasis by increasing epithelial-mesenchymal transition (EMT); conversely, knockdown of METTL3 significantly reduced the expression of Snail, a key transcription factor modulating EMT in cancer metastasis (48). Furthermore, through YTHDF2-dependent post-transcriptional silencing of suppressor of cytokine signaling (SOCS2), METTL3 was discovered to serve a pro-oncogenic role in human HCC by increasing proliferation and metastasis (49). Although some progresses have been made, it is still necessary to investigate the role of m^6^A modification on specific mRNA in normal physiological activities and abnormal pathological changes. Currently, the commonly used methods to detect RNA m^6^A methylation are m^6^A-seq, methylated RNA immunoprecipitation sequencing (MeRIP-seq), and photo-crosslinking-assisted m^6^A sequencing (PA-m^6^A-seq) (50, 51). **Figure 1** shows the process of DNA methylation and RNA methylation.

## 3 Mechanisms of nucleotide methylation in the regulation of fatty liver and steatohepatitis, liver fibrosis and cirrhosis, and HCC

Nucleotide methylation modifications can affect cell growth and homeostasis by promoting or repressing the expression of specific genes. Abnormal gene methylation may alter cellular physiology and functions, resulting in disease development. Nucleotide methylation has been linked to the development and progression of various chronic liver diseases, including fatty liver, steatohepatitis, liver fibrosis, cirrhosis, and HCC. In this section, we discuss how nucleotide methylation contributes to the etiology and progression of numerous liver illnesses by repressing or activating the expression of associated genes.

### 3.1 Mechanisms of nucleotide methylation in the regulation of fatty liver and steatohepatitis

DNA methylation and demethylation are involved in MAFLD and NASH. As liver inflammation and disease progressed, it was discovered that the total DNA methylation levels were considerably lower in the livers of MAFLD patients (52). The hypomethylated levels of protein kinase C epsilon (PRKCE) and sec14-likeprotein3 (SEC14L3) were related to body mass index (BMI), waist circumference, total triglycerides, high-density lipoprotein cholesterol, alanine aminotransferase and aspartate aminotransferase (53). In addition, a high-sugar diet increases nuclear 25-hydroxycholesterol (25HC) to activate DNMT1, which regulates cell lipid metabolism genes through DNA methylation, contributing to MAFLD and metabolic syndrome (54). Patients with NASH have greater methylation levels of the mitochondrial NADH dehydrogenase-6 (MT-ND6) gene than those with simple steatosis, which can lower mRNA expression levels. Of note, elevated methylation and transcriptional downregulation of MT-ND6 correlate with the severity of MAFLD (55).

M^6^A methylation and its regulators also play a regulatory role in the development of MAFLD and NASH. The m^6^A methylase METTL3 in liver tissue of T2DM patients was up-regulated, and METTL3 improved the expression level of fatty acid synthase (FASN) mRNA through m^6^A modification, which in turn promoted fatty acid metabolism while inhibiting insulin sensitivity of the liver. It is indicated that m^6^A and its regulators could participate in the occurrence of metabolic diseases by regulating fat metabolism (56). Mice overexpressing IGF2BP2 are more likely to develop steatohepatitis and progress to HCC. However, IGF2BP2-deficient mice show remarkable resistance to the diet-induced fatty liver *via* enhancing the expression of mRNAs encoding mitochondrial proteins, such as uncoupling protein-1 (UCP1) (57). Glucocorticoid receptor (GR)-dependent FTO transactivation and m^6^A demethylation on FTO mRNA promote lipid accumulation in hepatocytes; FTO knockdown dramatically attenuates dexamethasone-induced fatty liver in mice, further supporting the role of m^6^A on lipid synthesis. These results implicate FTO and m^6^A demethylation as key players in the pathogenesis of MAFLD (58). FTO can also regulate lipid synthesis and increase lipid accumulation in hepatocytes by promoting the maturation of sterol regulatory element binding protein 1c (SREBP1c) and enhancing the transcription of cell death-inducing DFFA-like effector c (CIDEC) (59). It has also been shown that FTO can stimulate inflammation in the liver by blocking m^6^A mRNA methylation of IL-17RA (60).

### 3.2 Mechanism of nucleotide methylation in the regulation of liver fibrosis and cirrhosis

Nucleotide methylation has been shown to exert a role in the initiation and progression of liver fibrosis and cirrhosis. Hepatic fibrosis is partly driven by the transdifferentiation of quiescent hepatic stellate cells (HSC) into active myofibroblasts; this process has been shown to significantly reduce the overall DNA methylation level, with over 20% of about 400 methylation regions being hypermethylated or hypomethylated (61). The DNA methylation reader methyl CpG binding protein 2 (MeCP2) can be recruited to the promoter region to inhibit transcription of peroxisome proliferator-activated receptor γ (PPARγ), thereby promoting the occurrence of liver fibrosis. PPARγ plays a negative role in regulating the transdifferentiation of myofibroblast into hepatic myofibroblast (62). The hypermethylation level of CpG dinucleotides within the promoter of the human PPARγ gene was found to be beneficial in distinguishing mild or severe fibrosis patients in a cohort analysis. This finding suggests that specific CpG islands’ methylation status may be an important predictor of liver fibrosis progression (63). A recent study reported that, compared to healthy controls, patients with cirrhosis and HCC had considerably more hypomethylated sites (64) and hypermethylated sites (65) among 27578 CpG motifs studied (66). These findings suggest that DNA methylation may contribute to liver fibrosis and cirrhosis, but the underlying mechanisms need further investigation.

RNA methylation also has its unique regulatory role for liver fibrosis. METTL3 was discovered to be highly elevated in HCC. By m^6^A methylation sequencing, one study identified SOCS2 as a target of METTL3-mediated m^6^A modification (49). In general, SOCS2 was hypermethylated in HCC and was associated with JAK-STAT pathway activation (67). Aside from its role in HCC, SOCS2 regulates liver fibrosis as well. Specifically, the LncRNA NEAT1/microRNA-129-5p/SOCS2 axis controls liver fibrosis in alcoholic steatohepatitis (68). Another study showed that acid-sensitive ion channel 1a (ASIC1a) promoted liver fibrosis by regulating miR-350 through METTL3-dependent m^6^A modification. Liver fibrosis patients and animal models had considerably higher levels of ASIC1a expression, and METTL3 was linked to ASIC1a’s promotion of liver fibrosis. METTL3-dependent m^6^A modification could regulate the synthesis of miR-350 by combining with DiGeorge syndrome critical region gene 8 (DGCR8). At last, mature miR-350 promoted liver fibrosis through PI3K/AKT and ERK pathways (69). Additionally, studies have demonstrated that RNA methylation modifications contribute to fibrosis in cirrhosis (70).

### 3.3 Mechanism of nucleotide methylation regulating HCC

HCC typically develops in patients with preexisting liver diseases, including hepatitis virus infection, alcoholic liver disease, and MAFLD, among which the prevalence of MAFLD is growing rapidly and is expected to be the leading cause of HCC. Studies have shown that nucleotide methylation can trigger changes in the HCC genome, which in turn cause dysregulation of related oncogene expression, and boost the initiation, progression, and metastasis of HCC.

The hypomethylation or hypermethylation of DNA methylation both can affect hepatocarcinogenesis and metastasis differently. Knockdown of osteopontin (OPN) can decrease the methylation of specific tumor suppressor genes (TSG), resulting in reduced DNA methylation and down-regulation of DNMT1 to inhibit tumor development and metastasis (71). Metabolic reprogramming is a typical feature of cancer cells. Glycolysis enzyme phosphoglycerate kinase 1 (PGK1) can catalyze 1,3-diphosphoglycerate to generate 3-phosphoglycerate and ATP simultaneously. Reduced PGK1 promoter methylation leading to higher expression is substantially associated with shorter overall survival and worse prognosis in HCC, as well as with a wide range of other malignancies (72). Data on 369 HCC patients’ genomics, methylomes, transcriptomes, proteomes, and clinical histories have been obtained and evaluated. It indicates that DNA methylation is connected with HCC prognosis (73). Angiogenesis plays an important role in the development, progression, and metastasis of HCC. MiR-378a-3p methylation induced by DNMT1 was shown to modulate the NF-κB signaling pathway, which promoted angiogenesis in HCC and was associated with a poor prognosis for HCC patients (74). In addition, methylation of mitochondrial fission regulator 2 (MTFR2) in HCC tissues causes aberrant expression and may contribute to HCC progression by influencing cell cycle progression, p53 signaling pathway, and DNA replication (75).

**Figure 2:**
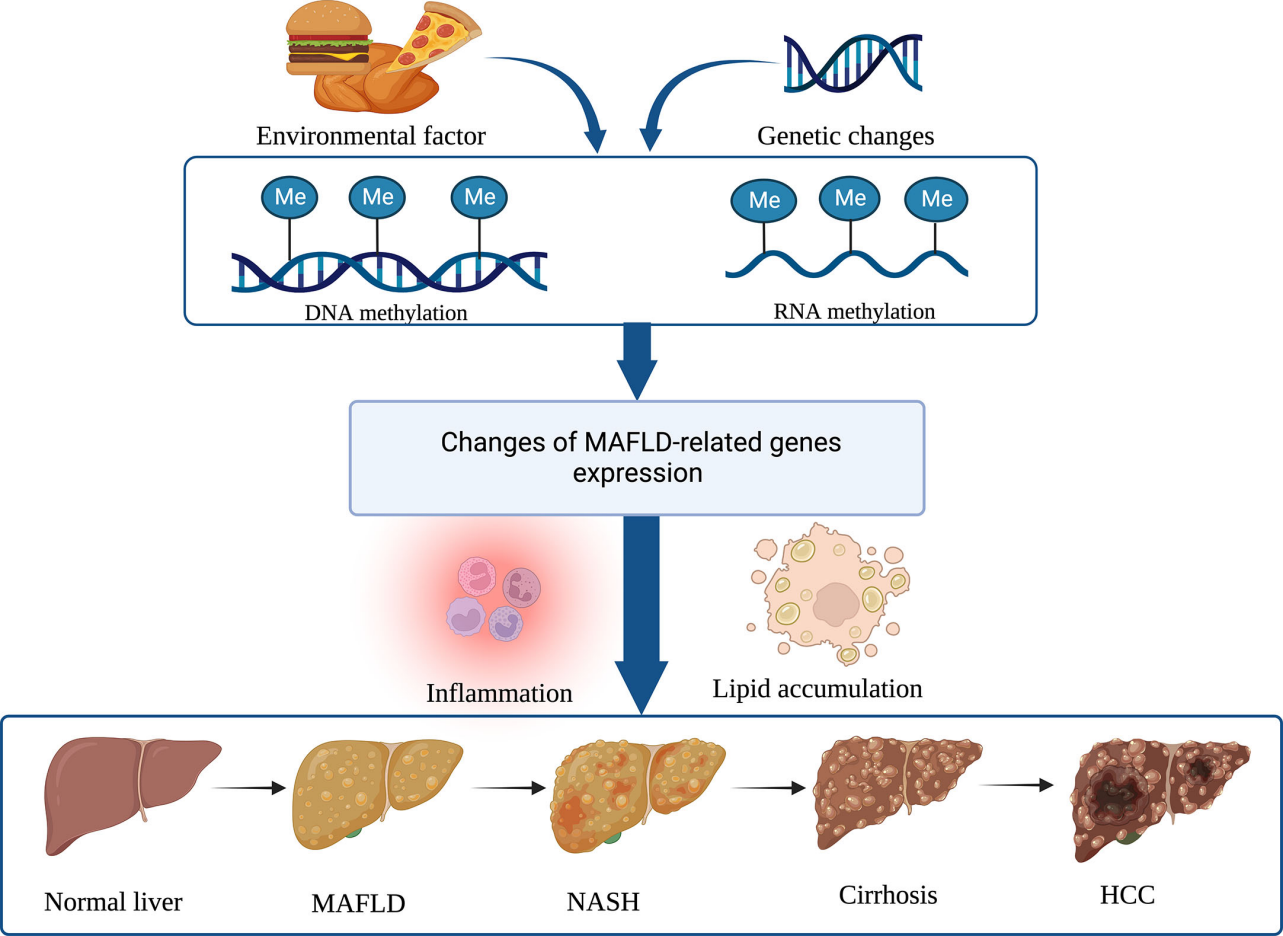
Nucleotide methylation regulates MAFLD and its progress. DNA and RNA methylations are the two main epigenetic modifications that alter gene expression involved in immune inflammation and lipid accumulation, and hence contribute to the development of MAFLD and its subsequent progression to NASH, cirrhosis, and HCC. (Created with BioRender.com).

M^6^A methylation is a well-known mRNA epigenetic modification involved in hepatocarcinogenesis. A high level of WTAP expression in HCC is linked to a worse prognosis. WTAP-directed m^6^A modification has been reported to increase HCC advancement *via* the HuR-ETS1-p21/p27 axis (76). Downregulation of RAD52 motif 1 (RDM1), a key regulator of DNA double-strand break repair and recombination, suppresses tumor growth in HCC, whereas deletion of RDM1 promotes HCC cell proliferation. As the data reveal, RDM1 can work with the tumor suppressor gene p53 to increase p53’s protein stability, suggesting that RDM1 has tumor-suppressive effects. However, overexpression of METTL3 can dramatically induce m^6^A modification of RDM1 mRNA and decrease its expression, which is involved in tumorigenesis (77). The ability of M^6^A mediated by YTHDF1 to accelerate the translation of snail mRNA suggests that m^6^A modification has a significant effect on HCC progression, and overexpression of YTHDF1 is associated with poor prognosis in HCC patients (48). Reportedly, YTHDF2 can directly bind to the m^6^A modification site of epidermal growth factor receptor (EGFR) to promote the degradation of EGFR mRNA in HCC cells. It is indicated that YTHDF2 may function as a tumor inhibitor by reducing the stability of EGFR mRNA in HCC cells, therefore suppressing cell proliferation and expansion (78). Nucleotide methylation’s potential impact on MAFLD progression is depicted in **Figure 2**.

## 4 Role of nucleotide methylation in the remodeling of the inflammatory immune microenvironment during the progression of MAFLD

Liver is capable of producing immune-inflammatory responses, and the pathogenesis and disease progression of MAFLD are closely related to the activation of immune-inflammatory responses (79). Increasing evidence confirms that inflammation and immune responses, including innate immunity and adaptive immunity, are crucial to the development of MAFLD. In particular, inflammation becomes an indispensable component of disease progression. Since nucleotide methylation is involved in stimulating and maintaining immune activation of inflammation, we subsequently discuss the role of nucleotide methylation in remodeling the IME during MAFLD progression.

### 4.1 Remodeling of immune cells in the inflammatory microenvironment of MAFLD by nucleotide methylation

The liver is home to a wide variety of immune cells, including macrophages, KCs, NKCs, and natural killer T cells (NKTs). T cells, B cells, and innate immune cells are all examples of acquired immune cells that could have a role in the onset and progression of MAFLD. The regulation of immune cells by nucleotide methylation may have a role in MAFLD.

DNA methylation is a key control factor of the phenotype of macrophages. DNA methylation of the promoter regions of 26 genes in CpG islands was identified and validated in carbon tetrachloride (CCl4)-induced liver fibrosis mice. Methylation of proline-serine-threonine phosphatase interaction protein 2 (PSTPIP2) was found to lead to mixed induction of hepatic classical macrophages (M1) and replacement macrophages (M2). The results showed that overexpression of PSTPIP2 suppressed M1 expression by inhibiting signal transducer and activator of transcription 1 (STAT1) activity and enhanced M2 expression by promoting STAT6 activity. Conversely, the knockdown of PSTPIP2 promoted M1 polarization and inhibited M2 polarization (80). MAFLD, obesity, and metabolic syndrome are closely related to the inflammatory response. Research reports that adipose-associated macrophages (ATM) in obese patients can shift from M2-type anti-inflammatory macrophages to M1-type pro-inflammatory macrophages (81), contributing to obesity-induced inflammation and IR. Additionally, macrophage infiltration is associated with the proliferation of preadipocytes; this process might cause adipogenesis and thus aggravate obesity (82). Pharmacological DNMT1 deficiency promotes selective macrophage activation by blocking PPAR promoter DNA methylation, and DNMT1-deficient mice show improved M2 differentiation, reduced macrophage inflammation, and ameliorated obesity-induced inflammation and IR (83). These results suggest that obesity-related DNA methylation is a critical factor in macrophage-induced inflammation.

CpG island hypomethylation has been reported as the predominant form of genomic DNA methylation associated with human NK cell activation, suggesting a crucial role for methylation in NK cell formation and function (84). In a global DNA methylation test of obese and T2DM patients, DNA methylation was discovered to be elevated in the participants’ B and NK cells. It may have contributed to the development of IR (85). In addition, the proliferation and function of macrophages and T cells are regulated in part by the ubiquitin-associated and SH3 domain-containing protein A (UBASH3A) and the tripartite motif protein 3 (TRIM3), both of which are hypermethylated in obese individuals (86).

METTL3 is specifically upregulated after M1 polarization in mouse macrophages, and further m^6^A methylation experiments demonstrate that METTL3 promotes M1-type macrophage polarization through methylating STAT1 mRNA (87). M^6^A is also involved in the regulation of T cell homeostasis and differentiation. Results show that METTL3 regulates CD4^+^ T cell proliferation and differentiation *via* targeting the IL-7/STAT5/SOCS signaling axis to maintain T cell immune homeostasis (46). The specific knockout of METTL3 in Tregs can lead to severe autoimmune diseases or even death in mice. Exploring the mechanism reveals that m^6^A modification can promote Treg-mediated suppression of immune cells *via* promoting degradation of SOCS mRNA in Treg cells. Decreased SOCS expression may subsequently activate the IL-2-STAT5 signaling pathway (88).

METTL14 deletion hinders B-cell development and significantly reduces the number of B cells. Results show that METTL14 deletion lowers IL-7-induced pre-B cell proliferation and induces aberrant gene expression linked to B cell development, suggesting that m^6^A methylation regulates early B cell development (89). In addition, YTHDF1-deficient DC cells can enhance tumor antigen presentation and the anti-tumor capacity of CD8^+^ T cells (90). The above studies suggest that m^6^A can regulate immune cells and play a regulatory role in inflammatory diseases, but most of these findings have not been considered in a liver-specific setting. Therefore, m^6^A modification of immune cells in regulating liver homeostasis and liver disease should be further explored.

KCs are macrophages in the liver with the function of phagocytosis, antigen processing, presentation, and producing a variety of inflammatory factors, including tumor necrosis factor-α (TNF-α) and IL-6 (91). KCs are divided into two broad phenotypes, the classically activated M1 macrophage, and the alternative M2 phenotype. The M1 phenotype is considered pro-inflammatory and capable of producing cytokines and chemokines, and M2 is considered anti-inflammatory. KCs in steatotic livers contain lipid droplets, recruit more lymphocytes, and release large amounts of pro-inflammatory factors that promote immune responses to facilitate MAFLD (92). The accumulation of KCs in the portal vein is an early event of MAFLD, preceding the aggregation of other immune cells (93). However, the role of the M2 phenotype of KCs in MAFLD and NASH is unclear (94).

DCs are also present in the liver as antigen-presenting cells. Studies have shown that DCs can limit sterile inflammation by removing apoptotic cells and necrotic debris (91). Still, a current study found that increased cDC1 of the DC subtype may promote CD8^+^ T cells, which can promote liver inflammation and injury in NASH, suggesting that cDC1 is a participant in the pathogenesis of NASH (95). Because the exact mechanism is not clear, continued research on the role of DCs in MAFLD is needed.

Invariant natural killer T (iNKT) cells are elevated in both NASH mouse models and NASH patients, where they secrete a variety of pro-inflammatory cytokines and boost OPN to exacerbate steatosis, NASH, and liver fibrosis, all of which contribute to the progression of MAFLD (96).

Neutrophil infiltration and neutrophil extracellular traps (NETs) are associated with chronic inflammation. in the early stages of fatty liver, NETs have been detected, indicating that neutrophil activation is a key factor in liver injury (97).

CD4^+^ T_H_ cells can differentiate into T_H_1, T_H_2, and T_H_17 cells with characteristic expression of interferon-γ (IFN-γ), IL-13, and IL-17, respectively (98), to participate in the process of NASH, liver fibrosis, and liver injury. T_H_17 cells are a newly discovered subgroup of CD4^+^ T cell characterized by the secretion of IL-17A, which plays a pathogenic role in promoting inflammatory diseases (65). It has been shown that T_H_17 cells are increased in NASH mouse models, especially a pro-inflammatory CXCR3^+^ T_H_17 cell subpopulation, driving NASH pathogenesis (99). In MAFLD, CD4^+^ memory T cell subsets are crucial in the transition from steatosis to fibrosis. After a high-fat diet (HFD), immunodeficiency mice implanted with human immune cells develop steatosis, liver inflammation, and fibrosis, along with increased liver and peripheral blood CD4^+^ memory T cells. Depleting human CD4^+^ cells in this model reduces liver inflammation and fibrosis, but not steatosis by secreting pro-inflammatory molecules such as IFN- γ and IL-17A (100).

CD8^+^ T cells mainly produce cytotoxic molecules such as IFN-γ, TNF-α, and perforin, and the number of hepatic CD8^+^ T cells is increased during NASH in mice and humans. Diet-induced NASH mouse models activate CD8^+^ T cells and NK T cells, which promote NASH and HCC through their interactions with hepatocytes (101).

B cells with roles in antigen presentation, immunoglobulin production, and cytokine secretion are involved in immune-mediated inflammatory responses. It has been demonstrated that patients with MAFLD have an accumulation of B cells in the liver and that B2 lymphocytes can develop into mature plasma cells. In addition, B lymphocytes can contribute to the occurrence of MAFLD through the secretion of cytokines, IgG, and in response to oxidative stress (102). In the mouse model of NASH, B cells exhibit pro-inflammatory effects through B cell receptor-mediated adaptive immune mechanisms and MYD88-dependent innate immune mechanisms. Moreover, intestinal microbial-induced inflammatory mediators in NASH liver contribute to B cell activation and disease progression (103).

**Figure 3:**
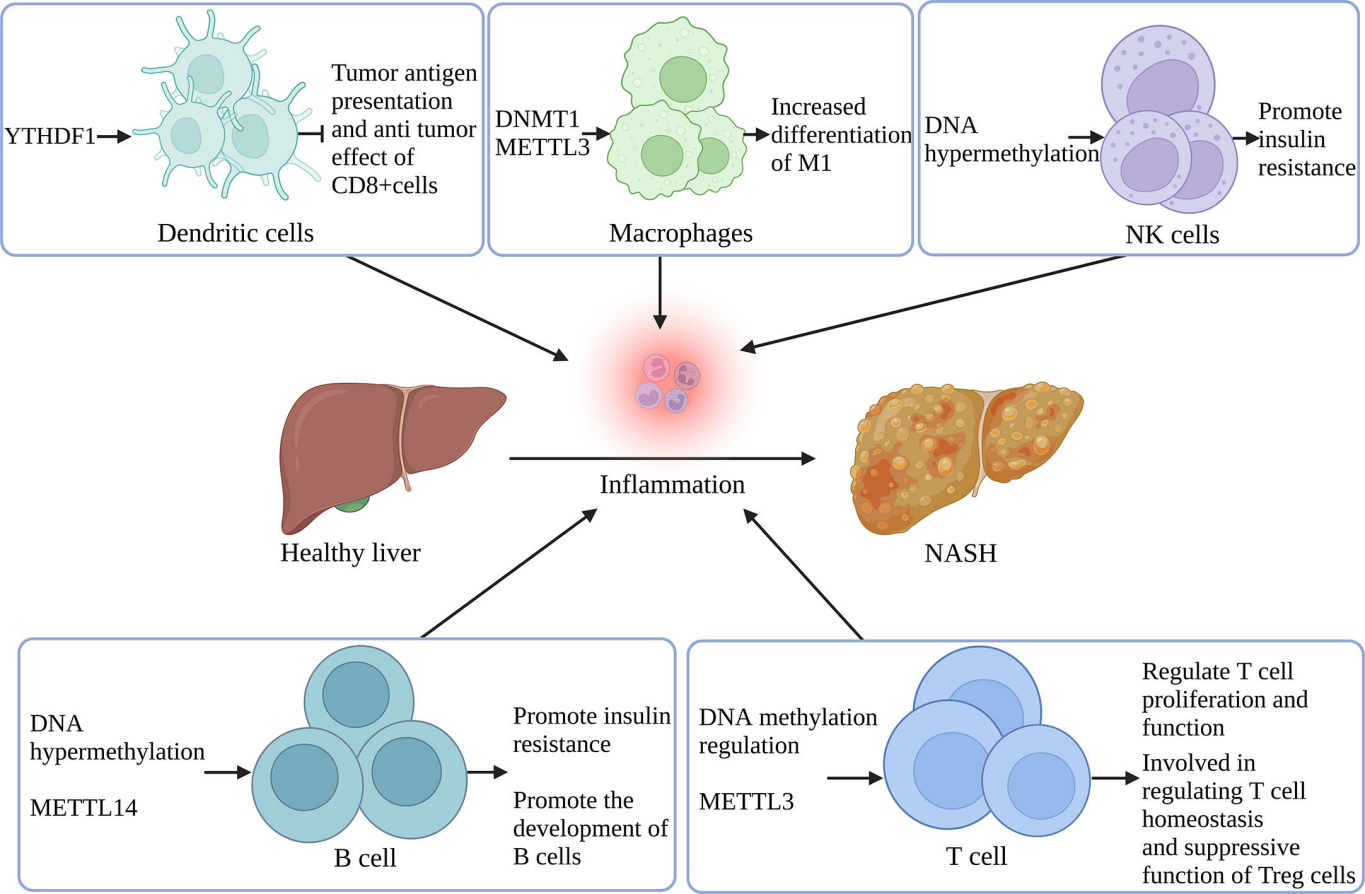
Remodeling of immune cells in the inflammatory microenvironment of MAFLD by nucleotide methylation YTHDF1 regulates tumor antigen presentation and anti-tumor effect in DCs. DNMT1 and METTL3 support M1 differentiation in macrophages. DNA hypermethylation in NK and B cells is associated with increased IR. METTL14 controls B cell development and maturation. DNA methylation also regulates T cell proliferation and function, and METTL3 participates in T cell homeostasis regulation and Treg’s immune suppression. (Created with BioRender.com).

It is thus clear that the remodeling of immune cells significantly influences MAFLD in the IME of the liver, and nucleotide methylation also has some regulatory effects on immune cells in MAFLD. However, due to the lack of studies on the special IME of the liver, the specific effects need further investigation. **Figure 3** shows the role of nucleotide methylation in remodeling immune cells during MAFLD.

### 4.2 Effect of nucleotide methylation on inflammatory factors in the inflammatory microenvironment of MAFLD

Nucleotide methylation affects inflammatory cytokines by regulating gene expression. DNA methylation regulates inflammatory cytokines, including IL-6, IL-11, and TNF-α, thus impacting the development of liver injury, diabetes, and obesity. It has been reported that the T-2 toxin, a highly toxic trichothecenes mycotoxins produced by Fusarium, can induce liver injury under inflammation. T-2 toxin can significantly increase the DNA methylase at the site of liver injury, inducing the expression of cytokines IL-11, IL-6, IL-1α, and TNF-α, thus aggravating the liver injury (104). A study has shown that the level of TNF-α gene methylation is related to the pathogenesis of T1DM (105). Another study discovered that obese males who lost weight had lower total TNF-α promoter methylation levels, suggesting that TNF-α promoter methylation can be utilized as a predictive biomarker of weight reduction (106). In obese patients, aberrant methylation of the IL-6 gene promoter is greatly enhanced, suggesting that it may be implicated in obesity’s pathophysiology (107).

RNA methylation has been confirmed to affect the IME by regulating a range of inflammatory factors and related signaling pathways. YTHDF2 knockout significantly increases the expression of IL-6, TNF-α, IL-1β, and IL-12 in lipopolysaccharide (LPS)-stimulated RAW 264.7 cells, and activates the NF-κB signaling pathway, hence promoting inflammatory response (108). Furthermore, METTL3 deletion in mouse T cells affects T cells’ homeostasis and differentiation *via* targeting the IL-7/STAT5/SOCS pathway (46). SOCS gene family expression is also found to be increased in METTL3-/- Treg cells and to reduce the suppressive function of Treg cells *via* IL-2/STAT5 (88).

### 4.3 Effect of nucleotide methylation on chemokines in the inflammatory microenvironment of MAFLD

Chemokines are the primary immune response-related drivers of hepatic immune cell infiltration during the immune response. Upstream regulators of chemokines include inflammatory cytokines including TNF, IL-1, IFN-γ, IL-17, and IL-6, which can promote chemokines’ transcription by stimulating nuclear factors and transcription factors (109). Additionally, as KCs in the liver are the primary producer of cytokines and chemokines in MAFLD, they can regulate hepatic inflammation *via* downstream proinflammatory signaling (110).

Nucleotide methylation may be involved in liver disease by influencing the expression of chemokines. In HCC samples showing vascular invasion, the methylation of C-X-C motif chemokine ligand 12 (CXCL12) gene is detecetd to be removed with a ratio of 22.1%, which could lead to increased expression. The CXCL12-CXCR4 axis expression is also significantly higher in HCC tissues, indicating that methylation deficiency of CXCL12 may be critical in vascular invasion of HCC (111). While preserving the activation and proliferation of CD4^+^ and CD8^+^ T cells and preventing the growth of colorectal tumors, METTL3 knockdown in colorectal cancer cells reduces myeloid-derived suppressor cell (MDSC) infiltration. Notably, METTL3 promotes the expression of BHLHE41 in an m^6^A-dependent manner and subsequently induces CXCL1 transcription to promote colorectal cancer (112). Tristetraprolin (TTP) is an immunosuppressive protein. TTP is upregulated in acute liver failure (ALF), and the mRNA stability of C-C motif chemokine ligand 2 (CCL2) and C-C motif chemokine ligand 5 (CCL5) is weakened by m^6^A modification, thus alleviating acute liver injury (113).

One study has found that CXC chemokine receptor 3 (CXCR3) is significantly elevated in the liver tissue of MAFLD patients and NASH mouse models. Moreover, induction of CXCR3 is related to the production of liver pro-inflammatory cytokines, T lymphocyte infiltration, endoplasmic reticulum stress (ER stress), and fatty acid synthesis (114). It has been discovered that the chemokine CCL2 is upregulated in the liver of MAFLD mice and that CCL2 enhances MDSC migration *in vitro*. MDSCs have been shown to inhibit T-cell immunity in tumor and inflammatory diseases. The findings imply that the CCL2-CCR2 pathway may contribute to the accumulation of MDSC in MAFLD, accelerating the disease’s pathogenesis and progression (115). CXCL10, a chemokine induced by IFN-γ, is associated with obesity and T2DM as well as being involved in the pathogenesis of NASH. Mice fed with methionine and choline deficiency (MCD) can develop NASH and increase the expression of CXCL10 in the liver. Consistent with the observation that inhibiting CXCL10 suppresses the development of steatohepatitis in mice, the results reveal that CXCL10 is implicated in steatosis *via* up-regulation of SREBP1c and liver X-activated receptor (LXR) (116). This finding suggests a potential function for CXCL10 in the development of NASH. A recent sutdy reported that hepatic CXCR6^+^ CD8 T cells are abundant in NASH mice and patients, and IL-5-induced downregulation of FOXO1 and upregulation of CXCR6 trigger hepatic CXCR6^+^ CD8 T cells to engage in self-attack, thereby harming the liver (117).

In overview, multiple immune cell populations and their secreted cytokines and chemokines are involved in the pathogenesis of MAFLD. The action of immune cells may cause the IME of MAFLD. At the same time, nucleotide methylation can also modulate immune cells, inflammatory factors, and chemokines; presumably, the two parts should be linked together. However, most studies on nucleotide methylation modifications of immune cells have not been performed in the liver-specific IME. Therefore, the specific effects and mechanisms of nucleotide methylation modifications of immune cells on the occurrence and development of MAFLD need to be studied in depth.

## 5 Effect of nucleotide methylation regulated metabolism on the IME in MAFLD

The liver is a crucial organ for glucose, lipid, and amino acid metabolism, and can maintain the body’s metabolic homeostasis. Metabolic disorders, brought on by a diet heavy in fat and sugar, can cause a variety of health problems, including lipotoxicity and cell damage. Constant cell death and damage set off a chain reaction of oxidative stress and KC activation that compromises the immune system and speeds up the progression of liver disease. It has been proved that nucleotide methylation is involved in various life processes, including glycolipid and amino acid metabolism. Therefore, this part will describe the effects of nucleotide methylation on the IME of MAFLD from the perspective of how it affects metabolisms.

### 5.1 Nucleotide methylation and regulation of lipid metabolism

The hallmark of MAFLD is the abnormal accumulation of triglycerides in liver tissue, which results from an imbalance between lipid acquisition, primarily derived from fatty acid uptake and lipid synthesis from scratch, and lipid clearance (16). Nucleotide methylation can change the balance of lipid metabolism by participating in fat formation, storage, and clearance. In addition, metabolism can affect the liver’s IME and promote the progression of MAFLD and HCC metastasis. Therefore, nucleotide methylation has the potential to impact the IME and the development of MAFLD *via* metabolic regulation.

Dysregulation of methylation of DNA is one of the major epigenetic changes that lead to MAFLD. DNA methylation regulates lipid metabolism by changing promoter methylation and participating in lipid synthesis, storage, and clearance. The methylated DNA donor is SAM, which can be obtained from food. One study has found that supplementing with methylated donors can alleviate the accumulation of triglycerides induced by an HFD in the liver. These results suggest that methyl-donor supplements may improve HFD-induced MAFLD by promoting hypermethylation of FASN genes (118). PPARs are nuclear receptors that can activate transcription factors, mainly PPARα, PPARβ, and PPARγ, and play physiological regulatory roles in the homeostasis of lipid and glucose metabolism, cell differentiation and development, and inflammation (119). The differentiation of preadipocytes can be promoted by the demethylation of the PPARγ promoter (120). Another study has found that an HFD during pregnancy increases PARAα methylation levels and reduces the expression of related proteins, resulting in obesity in offspring (121).

By decreasing methylation of the promoter for carnitine palmitoyl transferase 1 (CPT1A) and inducing hepatic lipid accumulation, an HFD leads to an increase in CPT1A, facilitating the transport of fatty acids into the mitochondria for β-oxidation (122). Increased methylation of ELOVL fatty acid elongase 2 (ELOVL2) in elderly fibroblasts reduces ELOVL2 expression, leading to ER stress and mitochondrial dysfunction and potentially accelerating aging *via* disruption of lipid metabolism (123). According to a gene cohort analysis, methylation changes of MAFLD-related genes are associated with lipid homeostasis, including apolipoprotein (APO) family for lipid transport, STAR-related lipid transfer domain 4 (STARD4) for cholesterol transport, STAR-related lipid transfer domain 10 (STARD10) for HDL metabolism, Niemann-Pick C1-Like 1 (NPC1L1) for cholesterol metabolism, and Solute Carrier family 47 member 1 (SLC47A1) for glucose and bile salt transport (124). Interestingly, the overall hypomethylation of circulating cell-free DNA in the plasma of individuals with MAFLD has been discovered. Decreased methylation of genes involved in lipid metabolism, such as Acyl CoA synthase long-chain family member 4 (ACSL4) and carnitine palmitoyltransferase1C (CPT1C), has been linked to the occurrence of MAFLD (125).

M^6^A can participate in the regulation of lipid metabolism through m^6^A regulatory proteins. Studies have investigated that genes with high m^6^A methylation levels in the HFD-induced fatty liver are mainly concentrated in pathways related to lipid metabolism (126). The m^6^A reading protein YTHDF2 recognizes and degrades the methylated mRNA of cyclin A2 (CCNA2) and cyclin-dependent kinase 2 (CDK2), and reduces the expression of CCNA2 and CDK2, thereby prolonging the cell cycle of adipocytes and inhibiting adipocyte differentiation (127). It has been reported that METTL3 inhibits bone marrow-derived stromal cell (BMSC) differentiation into adipocytes by targeting the JAK1/STAT5/C/EBPβ pathway in an m^6^A-YTHDF2-dependent manner (128). One study showed that the expression of lipid and cholesterol metabolism genes, including enoyl-CoA hydratase and 3-hydroxy acyl CoA dehydrogenase (EHHADH), FASN, and Sirtuin1 (SIRT1), is significantly decreased in METTL3 knockout mice, especially FASN (56). FTO promotes fatty acid synthesis and inhibits TG hydrolysis through RNA demethylation, thus promoting liver fat accumulation Analysis has shown that the decrease of m^6^A caused by the overexpression of FTO is associated with increased expression of genes related to lipid metabolism, including FASN, Stearoyl-CoA Desaturase1 (SCD1) and Monoacylglycerol acyltransferase 1 (MGAT1), and the decreased expression of genes related to lipid transport, including microsomal triglyceride transfer protein (MTTP), APOB and Hepatic Lipase (LIPC) (129). HFD-fed hepatocellular specific IGF2BP2 knockout mice have been found to exhibit reduced liver fatty acid oxidation, increased triglyceride accumulation, and moderate fatty liver disease, possibly due to significantly reduced expression levels of the CPT1A and PPARα (130). M^6^A methylation regulates the circadian rhythm of lipid metabolism. Loss of Bmal1 in the liver, an important component of the mammalian clock gene regulatory network, increases m^6^A methylation of PPARα mRNA and affects lipid metabolism, while METTL3 knockout can increase PPARα mRNA expression and reduce lipid accumulation *in vitro* (131).

**Figure 4:**
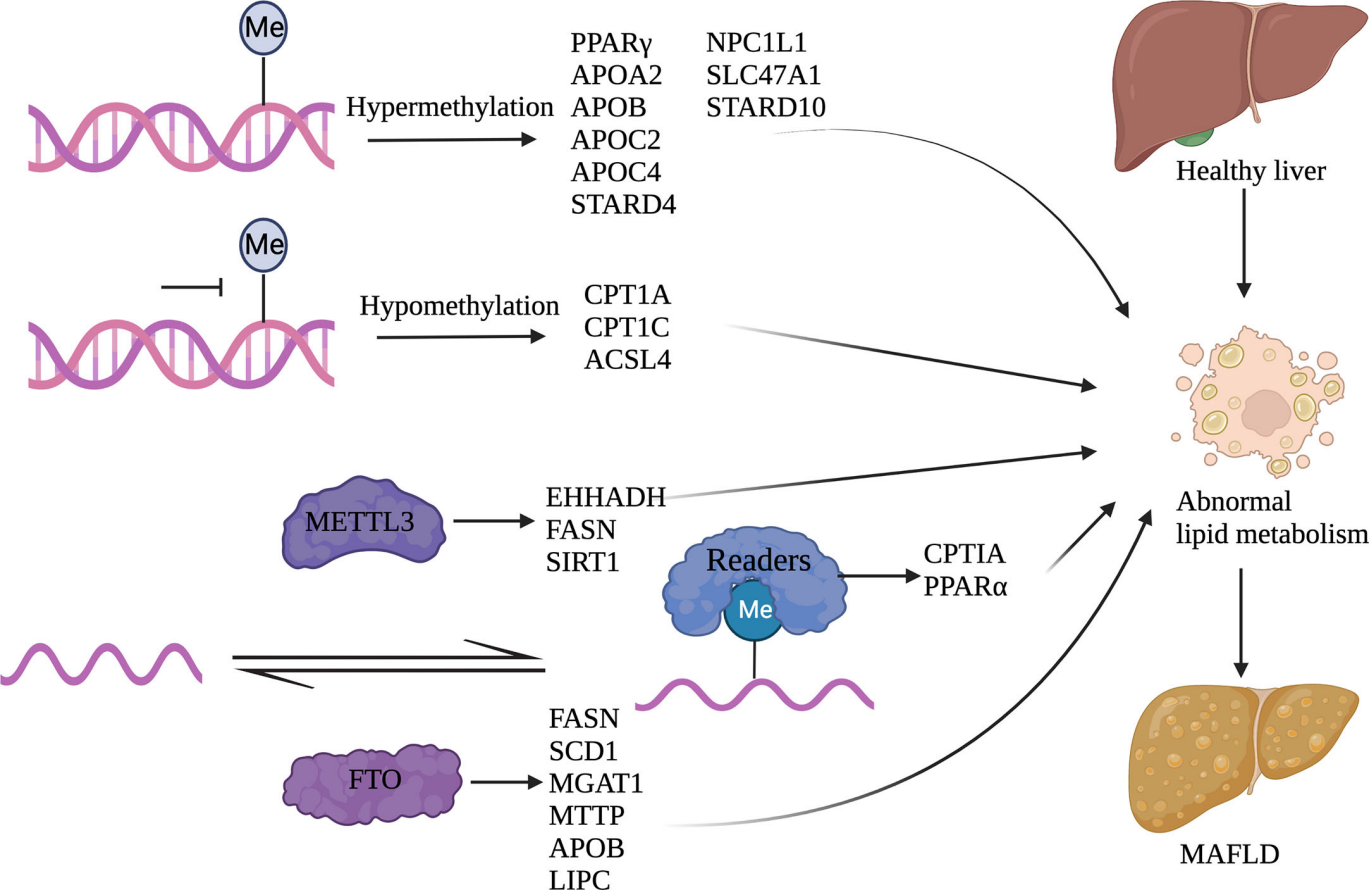
Nucleotide methylation and its regulation of lipid metabolism Abnormal lipid metabolism occurs in MAFLD. DNA methylation and RNA methylation can regulate gene expression involved in lipid metabolism. Among them, DNA hypermethylation genes include PPARγ, APOA2, APOB, APOC2, APOC4, STARD4, NPC1L1, SLC47A1, and STARD10; DNA hypomethylation genes include CPT1A, CPT1C, and ACSL4; METTL3 related genes include EHHADH, FASN, and SIRT1; FTO related genes include FASN, SCD1, MGAT1, MTTP, APOB, and LIPC; IGF2BP2 related genes include CPTIA and PPARα. (Created with BioRender.com).

Metabolic regulation and immune response are closely linked. Adipose tissue dysfunction involves the dysregulation of adipokine and cytokine release, thus promoting diabetes, inflammation, and atherosclerosis. Immune signals can also induce metabolic disorders resulting in hepatic steatosis (132). In the tumor microenvironment (TME), Treg function depends on the energy supplement pathway involved in lipid metabolism, and glucose uptake may promote the fatty acid synthesis of Tregs. Thus, there are close connections between the expansion of Tregs and lipid and glucose metabolism in the TME (133). **Figure 4** shows the effect of nucleotide methylation on lipid metabolism in MAFLD.

### 5.2 Nucleotide methylation and regulation of glucose metabolism

Glycolysis is the process of converting glucose to pyruvate to produce ATP and NADP after a series of metabolic reactions. Glycolysis has three rate-limiting enzymes: hexokinase (HK), phosphofructokinase (PFKM), and pyruvate kinase (PK), and insulin and glucagon are the main hormones that regulate blood glucose (134). Metabolites produced by glycolysis can enter the pentose phosphate, tricarboxylic acid cycle, lipid synthesis, and amino acid pathways. When glycolysis is dysregulated, the relevant metabolic pathways are also problematic (135).

As it is reported, IR is the main driving factor of MAFLD progress. Indeed, the essence of IR is that the peripheral tissues are less sensitive to insulin. In the IR state, the inhibition of insulin on hormone-sensitive lipase is weakened, thus promoting the continuous hydrolysis of fat, leading to the increase of free fatty acid (FFA). When the rise of FFA exceeds the compensatory capacity of the liver, it results in lipid metabolism disorder and hepatocyte steatosis. Elevated FFA levels in MAFLD patients negatively affect glucose metabolism, promote gluconeogenesis, and aggravate IR (136). Nucleotide methylation influences glucose metabolism by participating in glycogen synthesis and degradation, regulating islet development and differentiation, insulin secretion, and glucose metabolism-related pathways in MAFLD (137). Since glucose metabolism impacts the liver IME, nucleotide methylation has the potential to alter the liver IME by regulating glucose metabolism.

Recently, a comprehensive DNA methylation analysis was conducted on islets from T2D and non-diabetes. 276 CpG islands associated with 254 gene promoters were found to show significant differences in DNA methylation in T2D islets (138). It has been determined that hypomethylation of genes involved in hepatic glycolysis and IR leads to increased levels of expression products of genes participating in hepatic glycolysis and gluconeogenesis, which contributes to the development of obesity, IR, and T2DM (139).

DNA methylation may serve a role in the pathogenesis of MAFLD by modulating glucose metabolism, as it can influence enzymes involved in glycolysis. HFD in obese rats was discovered to cause aberrant DNA hypermethylation in the promoter regions of the liver glucokinase (GCK) and L pyruvate kinase (LPK) genes, leading to decreased production of GCK and LPK and an effect on blood glucose content (140). The Warburg effect, characterized by increased aerobic enzymes and lactate generation, is commonly seen in tumor cells. By modulating critical glycolysis enzymes, DNA methylation can directly regulate glycolysis and influence the Warburg effect (141). PK can be subdivided into PKM1 and PKM2 due to selective splicing. It has been found that PKM2 stimulates the Warburg effect by inducing DNA methylation, specifically related to the binding of DNA methylation-mediated brother of the regulator of imprinted sites (BORIS) at the PK exon. When DNA methylation is inhibited, glycolysis and the Warburg effect are both downregulated (142). One research reported that Hexokinase HK2 was upregulated in malignant glioma due to DNA methylation, and this upregulation was followed by increased aerobic glycolysis and tumor proliferation (143).

It is generally accepted that M^6^A is vital for maintaining glucose homeostasis, and dynamic changes in m^6^A modification alter the expression of signaling molecules and metabolism-related genes, leading to the development of metabolism-related diseases such as obesity, diabetes mellitus, and MAFLD. FTO, an m^6^A demethylase, is widely expressed in a variety of tissues and is involved in the development of metabolic diseases. It has been shown that FTO-dependent m^6^A modification regulates glucose metabolism by controlling the FTO-forkhead box O1 (FOXO1) axis in hepatic gluconeogenesis (144). METTL3 overexpression induced by an HFD exacerbates hepatic metabolic disorders and IR in hepatocytes. In contrast, METTL3 knockdown significantly improves HFD-induced metabolic disorders by slowing weight gain, reducing lipid accumulation, and improving insulin sensitivity (145). The expression of MELLT3 and the level of m^6^A methylated RNA are increased in liver tissues of T2DM patients and mice with HFD. METTL3 knockout could reduce the expression and m^6^A methylation levels of FASN, thus inhibiting the metabolism of fatty acids (56). Similarly, the m^6^A RNA reading protein IGF2BP2 promotes islet β-cell proliferation and insulin secretion by enhancing pancreatic and duodenal homeobox 1 (PDX1) expression (146).

Glucose metabolism and the hepatic IME are also significantly associated. Hepatic steatosis and persistent inflammation in patients with MAFLD affect the normal glucose metabolism process and promote IR and T2DM. Correspondingly, the presence of IR will eventually act on the inflammatory pathway, resulting in MAFLD and forming a vicious cycle. In the liver TME, PKM2 expression and aerobic glycolysis of pro-inflammatory immune cells are increased, promoting the infiltration of various immunosuppressive cells. Meanwhile, PKM2 knockdown inhibits the proliferation, migration, and invasion of HCC cells. These findings support the hypothesis that PKM2 facilitates HCC progression by creating an immunosuppressive microenvironment (147). Additionally, enhanced glycolysis often results from the increased expression of glycolytic-related enzymes in mononuclear macrophages that have infiltrated HCC tumors and peritumoral tissues. Glycolysis induces the expression of programmed cell death 1 ligand 1 (PD-L1) in monocytes *via* the PFKFB3-PD-L1 axis, thereby inhibiting the cytotoxic activity of T lymphocytes against tumor cells and promoting the progression of HCC (148).

### 5.3 Nucleotide methylation and regulation of amino acid metabolism

In addition to producing proteins, peptides, and other molecules needed for life, amino acid metabolism also allows for their decomposition *via* deamination and transamination, as well as their conversion into sugars, lipids, and even some non-essential amino acids. Many studies have found a link between amino acid metabolism and MAFLD (149). Nucleotide methylation exerts a specific influence on amino acid metabolism, and thus participates in the occurrence and progression of MAFLD by influencing amino acid metabolism.

Amino acid metabolism plays a particular role in the occurrence and development of MAFLD. Researchers have found that genes involved in glycine biosynthesis, especially alanine glyoxalate aminotransferase 1 (AGXT1), are suppressed in the livers of both humans and animals with MAFLD, suggesting that impaired glycine metabolism may be an etiological factor in this disease. A glycine-containing tripeptide can significantly improve HFD-induced NASH mice, reducing circulating glucose and lipids, and improving steatohepatitis (150). The enhanced IR and protein catabolism seen in obese and MAFLD patients is thought to account for their high plasma amino acid concentrations, notably in branched-chain amino acids (151). It has been demonstrated that supplementation with branched-chain amino acids may have a positive impact on the improvement of glucose metabolism, reduction of fat accumulation in skeletal muscle, and prognosis in patients with cirrhosis (152). Furthermore, the serum glutamate to glutamine ratio is correlated with myofibroblast density and severity of liver fibrosis in NASH progression, suggesting that glutamate plays a critical role in amino acid metabolism in the liver (153). Another finding is that phenylalanine can promote IR by inactivating insulin receptor beta (IRβ). This has been shown in animals fed a diet rich in phenylalanine, who thereafter develop IR and type 2 diabetes symptoms (154).

## 6 Clinical translational study of nucleotide methylation and MAFLD

Currently, there are no effective drugs for the clinical treatment of MAFLD. Simple lifestyle intervention cannot alleviate the disease for patients progressing to NASH, cirrhosis, and HCC. Nucleotide methylation-induced epigenetic changes have a specific regulatory role in MAFLD and its development into HCC. Its potential reversibility provides new ideas for developing new biomarkers and drugs for treating MAFLD and HCC.

DNA methylation biomarkers of HCC help to improve the diagnostic accuracy of HCC and effectively predict prognosis. The overall 5mC DNA methylation and the oxidized derivative of 5mC, 5-formylcytosine (5fC), are significantly reduced in the early stages of HCC. The genomic DNA content of 5mC and reduced 5fC levels are related to poor prognosis in HCC patients and may also be potential biomarkers predicting prognosis, whereas dynamic variations in 5mC can be utilized to differentiate the staging of HCC (155). CXCL2, an immune-related chemokine whose regulatory mechanism is related to DNA methylation, was significantly down-regulated in tumor tissues, and tumors with higher CXCL2 expression contained more multiple tumors than those with lower expression (156). P16 is a tumor suppressor gene associated with the cell cycle, and hypermethylation of the promoter region can lead to cancer development, so abnormal methylation of the p16 promoter may be a promising marker for detecting HCC (157). Keratin 19 (K19) promoter methylation is significantly associated with K19 deficiency, and K19-expressing HCC patients have a worse prognosis than K19-deficient patients. It is speculated that K19 expression is a potential predictor of poor prognosis in HCC patients (158).

The inositol 1, 4, 5-trisphosphate receptor (ITPR3) is absent or under-expressed in hepatocytes from the normal liver. However, multiple regions of the ITPR3 gene are hypomethylated in HCC samples from three separate patient cohorts, and higher ITPR3 expression is correlated with shorter survival times in HCC patients (159). Acyl-CoA dehydrogenase (ACADS) has been found to inhibit HCC carcinogenesis and metastasis. The methylation of ACADS, which is influenced by DNMTs, was shown to be upregulated in HCC, while DNMTs knockdown increased ACADS expression, implying that it could be helpful as a diagnostic or prognostic marker (160). HCC suppressor 1 (HCCS1) promoter methylation is frequently detected in the serum of HCC patients, and it is more likely to be seen in patients with advanced tumor lymph node metastasis (161). CCAAT/enhancer-binding protein-beta (C/EBPβ) enhancer hypomethylation, which activates C/EBPβ expression, is associated with poor prognosis. It is also discovered that the deletion of C/EBPβ enhancer reduces the tumorigenicity of HCC (162). Glycerol-3-phosphate dehydrogenase 1 like (GPD1L) expression is substantially increased in HCC patients with vascular invasion compared to those without; meanwhile, high GPD1L expression is linked to poor overall survival and HCC recurrence (111). All of the above mentioned have the potential to become biomarkers for HCC in connection with diagnosis, treatment or prognosis, as shown in **Table 1**.

**Table 1:** Biomarkers of nucleotide methylation in HCC.

Nucleotide methylation types	DNA methylation biomarker	Function	Potential biomarker role in HCC	References
DNA methylation	CXCL2	Immunochemokine	Diagnosis and treatment of HCC	(156)
	p16	Cancer suppressor gene involved in cell cycle regulation	HCC detection marker	(157)
	ACAD	Involved in tumor proliferation and metastasis	Diagnosis and prognosis of HCC	(160)
	HCCS1	HCCS 1	Diagnosis and prognosis of HCC	(161)
	K19	Vascular invasion, poorly differentiated tumors, tumor recurrence after resection	Poor prognosis in patients with HCC	(158)
	ITPR3	Cell proliferation	Pathogenesis of HCC	(159)
	C/EBPβ	Early tumorigenesis	Poor prognosis in patients with HCC	(162)
	GPD1L	Vascular invasion	Poor outcomes in patients with HCC	(111)
RNA methylation	METTL3	Promote HCC progression through YTHDF2-dependent SOCS2 posttranscriptional silencing	Poor prognosis of patients with HCC	(49)

Promoter hypermethylation is a biomarker of HCC, so it is also a potential therapeutic target. HFD mice can be detected DNA damage, DNA genome-wide hypomethylation, and promoter hypermethylation. Vitamin E treatment in HFD mice has been shown to reduce DNA damage and significantly reduce methylation of the same CPG in HFD mice given vitamin E compared to HFD controls (64). Recent research suggests that targeting epigenetic regulators can control the progression of HCC. 5-azacytidine (5-AZA) is a first-generation DNMT inhibitor (DNMTi) that binds to newly synthesized DNA, binds irreversibly to DNMT1, and induces DNMT1 degradation and DNA demethylation. The demethylated compound 5-AZA at non-cytotoxic doses promotes the anticancer response by inhibiting the tumorigenicity of HCC cells (163). Guadecitabine (SGI-110), a second-generation DNMTi, impedes tumor growth and inhibits angiogenesis in a xenograft HCC HepG2 model. Still, it fails to prevent liver fibrosis and inflammation in a mouse model of steatohepatitis (164). The results suggest that SGI-110 reverses most of the aberrantly transcribed genes in HCC tumors and stimulates the innate immune response, and it is hypothesized that SGI-110, in combination with other cancer therapeutics, may be more effective in increasing sensitivity to immune checkpoint therapy and resensitizing drug-resistant tumor cells (165).

Decitabine (DAC), an inhibitor of DNA methylation transferase, is able to induce CXCL10 expression in tumor cells and suppress anti-tumor T-cell responses and hepatoma proliferation when administered to HCC mice (166). Doxorubicin (DOX) is a DNMT1 inhibitor that impacts telomerase activity and leads to the induction of apoptosis, thereby killing cancer cells (167). Zebularine (ZEB), a DNA methyltransferase inhibitor, can bind preferentially to DNA, reduce DNA methyltransferase levels, and alter p16 gene expression and demethylation, so displaying a more effective tumor cell growth suppression (168). The growth of tumors that overexpress the human organic cation transporter-1 (hOCT1, encoded by the SLC22A1 gene) is inhibited by sorafenib, although hOCT1 expression is low in HCC, and hOCT1 expression is negatively correlated with SLC22A1 promoter methylation. Therefore, increasing hOCT1 expression is a possible approach to improve the sensitivity of HCC to the pharmacological effects of sorafenib (169).

Since m^6^A RNA methylation has a significant role in the progression of different liver diseases, targeting m^6^A RNA methylation to treat liver diseases may provide potential therapeutic approaches. Several inhibitors targeting FTO, such as entacapone and meclofenamic acid (MA), have been identified. Entacapone has been shown to affect liver gluconeogenesis and adipose tissue by acting on the FTO-FOXO1 regulatory axis (144), and administration of entacapone reduces body weight and decreases fasting glucose concentrations in diet-induced obese mice. MA competes with FTO binding for m^6^A-containing nucleic acids (170) and prevents the increase of total triglycerides in oleic acid/dexamethasone (OA/DEX) (58). The yellow polyphenolic compound curcumin can affect the expression of METTL3, METTL14, ALKBH5, FTO, and YTHDF2 mRNA and increase m^6^A modification in the piglet liver. It could attenuate LPS-induced liver injury and hepatic lipid metabolism disorders by changing m^6^A RNA methylation (171). In human HCC, METTL3 overexpression is associated with poor prognosis in HCC patients (49), so the expression of m^6^A regulators may be a potential biomarker for prognosis prediction in HCC patients. METTL3 is associated with the occurrence and maintenance of acute myeloid leukemia (AML), so one study has explored whether STM2457, as a small molecule specific inhibitor of METTL3 enzyme, targeting METTL3 enzyme activity has anti-leukemia therapeutic potential. The results showed that STM2457 could reduce m^6^A levels and lead to abnormal mRNA translation, prevent the progression of AML, and reduce key leukemia stem cells *in vivo*, demonstrating that the targeting of RNA modifying enzymes is a promising anticancer therapy pathway (172). Immune checkpoint therapy is currently a new hot topic in cancer therapy. By targeting programmed death 1 (PD1) in cytotoxic T cells or PD-L1 in cancer cells, immune checkpoint therapy activates the adaptive immune system to clear cancer cells. It is found that knockdown of YHTDF1 significantly improves the anti-tumor response to anti-PD-L1 therapy (90), so targeting m^6^A regulatory factors may be a potential therapeutic approach to improve the immune potential therapeutic approach for checkpoint therapy. As a key m^6^A reading protein, IGF2BP1 has been implicated in tumorigenesis. It has been found that IGF2BP1 knockdown can induce apoptosis of cancer cells, significantly activate immune cell infiltration, and also reduce the expression of PD-L1 in HCC. A drug called cucurbitacin B (CuB) was found to directly target IGF2BP1 and block its recognition of m^6^A, resulting in the induction of apoptosis of cancer cells *in vivo*, the activation of immune cells into the TME and the reduction of PD-L1 expression in HCC, demonstrating substantial anti-HCC effects and improving the TME (173). Including the above studies, there are also related DNA methylation drugs in **Table 2**.

**Table 2:** Drugs related nucleotide methylation for the treatment of HCC.

Nucleotide methylation types	Drugs	Substance of drug	Mechanisms of action	References
DNA methylation	5-AZA	Nucleotide analogues	Reduce DNA methylation by inhibiting DNA methylation transferase and re-expression of epigenetic silenced genes to promote anti-cancer effect	(163)
	SGI‐110, a novel second‐generation DNA methylation inhibitor	A second-generation DNA methylation inhibitor formulated as a dinucleotide of DAC and deoxyguanosine	Demethylate the promoter of tumor suppressor gene and inhibit tumor growth and angiogenesis	(164)
	5-AZA-DC: DAC	The DNA methyltransferase inhibitor	Directly incorporate DNA, cause DNA hypomethylation and activate silent TSG	(166)
	DOX	An inhibitor of DNMT1 and a hydroxyl derivative of daunorubicin	Affect telomerase activity, induce apoptosis, and kill cancer cells	(158)
	Vitamin E	Lipid-soluble vitamin	Reduce DNA damage, regulate expression of DNMT1 and reduce oxidative stress	(64)
	ZEB	A nucleoside analog of cytidine and a new DNMTi	Reduce DNA methylation by inhibiting DNA methylation transferase and prevent the occurrence of remethylating	(168)
RNA methylation	Entacapone	A chemical inhibitor of FTO	Bind directly to FTO and inhibits FTO activity *in vitro*, reducing body weight and fasting glucose concentration	(144)
	MA	A non-steroidal, anti-inflammatory drug	Compete with FTO binding for the m^6^A-containing nucleic acid	(170)
	Curcumin	A natural compound with good anti-inflammatory and anticancer properties	Alleviate LPS-induced lipid metabolism disorders in liver through m^6^A RNA methylation modification	(171)
	CuB	A class of tetracyclic triterpenoids isolated from Cucurbitaceae	Directly target IGF2BP1 and block its recognition of m^6^A, thereby inducing apoptosis of cancer cells *in vivo*, activating immune cells into the TME and reducing PD-L1 expression in HCC	(173)

M^6^A regulators are associated with tumor resistance, and patients exhibit higher tolerance to tyrosine kinase inhibitor (TKI) during TKI therapy because of reduced m^6^A due to FTO overexpression in leukemia cells (174). Cells lacking METTL3 show higher sensitivity to anticancer agents such as gemcitabine, 5-fluorouracil, cisplatin, and radiotherapy (175). However, to develop assays that can identify cancer-specific m^6^A modifications for early diagnosis and create new specific inhibitors that target m^6^A or m^6^A regulators, more research is required to investigate the role of m^6^A modifications and m^6^A regulators in the development of HCC and their potential as therapeutic targets.

In clinical treatment, epigenetic drugs can also be used in combination with traditional anti-liver cancer drugs, improving the efficacy and reducing the side effects of conventional anticancer drugs. Low-dose SGI-110 pretreatment with oxaliplatin can produce enhanced cytotoxicity. Compared with oxaliplatin alone, the combination of SGI-110 and oxaliplatin can significantly delay tumor growth in mice (176). Due to its severe toxicity and adverse reactions, cisplatin has a poor effect on HCC, but curcumin can improve the sensitivity of HCC to chemotherapy drugs. Studies have shown that cisplatin combined with curcumin in mouse HCC H22 and human HCC HepG2 xenograft models shows better antitumor effects and reduces side effects compared with monotherapy (177).

With the deepening of genomics and metabolomics research on the etiology of MAFLD and HCC, targeted drugs targeting the epigenetics related to MAFLD are also under continuous research. For patients who cannot alleviate the disease through lifestyle regulation, targeted drugs will be an effective way to improve or even reverse liver histology. Multigroup treatment will effectively prolong the survival time of patients with advanced MAFLD, improve the quality of life, and avoid related complications.

## 7 Discussion and conclusion

The accumulation of lipids in the liver can cause steatosis and even lipotoxicity. With the ER stress and mitochondrial dysfunction caused by steatosis, liver cells are damaged, and a large number of inflammatory factors are secreted, leading to damage or death of liver cells, which may further develop into NASH, liver fibrosis, and HCC (178). As an immune organ, the liver has many kinds of immune cells. At the initial stage of liver cell damage, residential DCs, KCs, and other immune cells release many proinflammatory cytokines and chemokines in response to stress. Chemokines will promote further infiltration of inflammatory cells, intensifying the inflammatory response, thus leading to cell apoptosis and inducing NASH (179). Damage-associated molecular patterns (DAMPs) are released by damaged or dead hepatocytes, continuing to induce strong inflammatory responses; these DAMPs include mitochondrial damage-related molecular patterns (mito-DAMP), in which mitochondrial DNA directly activates KCs, setting off an inflammatory cascade and driving the occurrence and development of liver fibrosis (180). An imbalance of the intrinsic immune regulatory system also has a facilitative role in the development of MAFLD, contributing to hepatic steatosis, IR, and fibrosis (181).

Epigenetic changes are closely linked to the pathogenesis of MAFLD. Epigenetic modifications are involved in lipid metabolism, IR and ER stress, mitochondrial damage, oxidative stress, and inflammation, which work in concert to induce MAFLD. Nucleotide methylation can regulate MAFLD from both aspects of immunity and metabolism. Nucleotide methylation can participate in pathways related to lipid metabolism, glucose metabolism, and amino acid metabolism; meanwhile, the metabolic environment can influence immune cells, cytokine release, and regulate signaling pathways such as the IL-2-STAT5 signaling pathway. Therefore, nucleotide methylation can regulate the IME of the liver by indirectly altering metabolism, thus participating in the development and progression of MAFLD. As MAFLD progresses, nucleotide methylation remodels the IME by regulating immune cells like macrophages, NK cells, B cells, and T cells, inflammatory factors like IL-11, IL-6, IL-α and TNF-α, and chemokines like CXCL2, CXCL3, CXCL10, as well as associated signaling pathways that are crucial to MAFLD pathogenesis.

Nucleotide methylation is an important determinant of MAFLD and is strongly associated with the progression of MAFLD, including NASH, liver fibrosis, cirrhosis, and HCC. Nucleotide methylation markers have the potential as therapeutic targets and staging biomarkers. They are expected to identify patients with early and progressive MAFLD by developing noninvasive operational screening techniques, such as blood-derived biomarkers (182). Because the nucleotide methylation or demethylation state is reversible, a shift from an HFD to a regular diet and weight loss after bariatric surgery can also partially normalize the DNA methylation profile in MAFLD. However, for patients whose disease cannot be alleviated through lifestyle modification, targeted drugs targeting nucleotide methylation may be a useful way to improve or even reverse liver histological status in addition to bariatric surgery. Improving the screening, diagnosis, and treatment of patients with NASH-related HCC can, to some extent, reduce the suffering of patients with MAFLD and delay the development of the disease, thus bringing benefits to patients with MAFLD (12, 183). Drugs targeting nucleotide methylation have opened up new ideas for MAFLD treatment. However, their role in liver disease needs to continue to be explored and these drugs’ specificity and side effects should be clarified. Therefore, more studies on these drugs are needed *in vitro* and *in vivo*. And combining drugs targeting nucleotide methylation with existing medical treatments, such as immune checkpoint blockade, chemotherapy, or radiotherapy, may lead to more effective treatments. In conclusion, a better understanding of the mechanisms of nucleotide methylation in the pathogenesis of MAFLD will lead to better diagnostic, prognostic, and therapeutic interventions, and promise great advances in personalized medicine.

## Author contributions

Conceptualization, SJ and QL. writing original draft preparation, NZ. writing review and editing, SJ, QL, XT, HW, DZ, and TY. visualization: NZ and CL. supervision: SJ and QL. funding acquisition: SJ. All authors contributed to the article and approved the submitted version.
